# Comparative molecular dynamics simulation analysis of G20 and C92 mutations in c-di-GMP I riboswitch and the wild type with docked c-di-GMP ligand

**DOI:** 10.6026/97320630017721

**Published:** 2021-08-31

**Authors:** Priyanka Kumari, Anup Som

**Affiliations:** 1 Centre of Bioinformatics, Institute of Interdisciplinary Studies, University of Allahabad, Prayagraj – 211002, India

**Keywords:** Molecular docking, MD simulation, Riboswitch-ligand complexes, Binding free energy, hydrogen bonding

## Abstract

Riboswitch, a bacterial regulatory RNA consists of an aptamer (specific ligand binding unit) and an expression platform (gene expression modulation unit), which act as a potential drug target as it regulates critical genes. Therefore, it is of interest to glean information on the binding of c-di-GMP ligand to mutated conserved G20 and C92 residues of cyclic diguanosine monophosphate I (c-di-GMP I) riboswitch using molecular dynamics simulation. The result shows that the binding energy of wild/native type riboswitch-ligand complex (3IRW) is lower than the mutant complexes suggesting that the binding affinity for c-di-GMP ligand decreases in case of mutant riboswitches. The hydrogen bonding interactions analysis also showed a high number of hydrogen bonds formation in the wild type riboswitch-ligand complex as compared to the mutant complexes illustrating stronger interaction of ligand to wild type riboswitch than the mutants. The simulation result shows that the mutations affected riboswitch-ligand interactions. The residues G14, G21, C46, A47, and U92 were identified as the key residues which contributed effectively to the binding of c-di-GMP I riboswitch with the natural ligand.

## Background:

Riboswitches, the bacterial molecular switches regulate gene expression in many bacterial systems. They are structural RNAs present generally at the 5' end of mRNAs which binds to specific ligand/metabolite and bring about the conformational changes resulting in modulated gene expression according to changes in the physiology of the bacterial system [1,2]. Thus riboswitches are considered as potential drug target against pathogenic bacteria [3,4]. One such riboswitch is c-di-GMP I riboswitch that binds to c-di-GMP ligand and is pivotal in regulation of gene expression and maintenance of cellular homeostasis caused by alteration in physiology. These riboswitches regulate genes, which are involved in several metabolic processes such as mobility, quorum sensing for pathogenic bacteria such as Vibrio cholerae [5]. Therefore, the role of c-di-GMP riboswitch attracts the researchers to explore and modulate its mechanism of action at molecular and dynamical level for the development of antibacterial drugs. Mutation studies have became essential to evaluate significance of each residue and their role in the binding pocket, here we study the effect of mutated residues on binding energy of ligand and receptor and changes in their interactions (hydrogen bonds and hydrophobic bonds) in order to find the exact atom behaviour for the corresponding change at molecular level which adds new knowledge [6]. The crystal structures of both wild and mutant c-di-GMP I riboswitches bound to c-di-GMP ligand were reported earlier. The crystal structure of native c-di-GMP I riboswitch suggests a three helical structure (P1, P2 and P3) and a fork like three-way junction J1/2, J2/3 and J1/3 connecting these helices. The junction nucleotides form the catalytic pocket that binds c-di-GMP ligand to it. The wild type and mutant c-di-GMP I riboswitch share highly conserved primary and secondary structure [7]. It was also observed that adenine 47 (A47) is phylogenetically conserved in all the class I c-di-GMP riboswitches while G20 and C92 were less conserved. Therefore, it is critical to gain information on the binding of docked c-di-GMP ligand to mutated conserved G20 and C92 residues of cyclic diguanosine monophosphate I (c-di-GMP I) riboswitch using molecular dynamics (MD) simulation.

## Materials & Methods:

### Initial Structure Preparation:

Atomic co-ordinates for wild type (PDB Code: 3IRW) and mutated systems (PDB Codes: 3MUM, 3MUR, 3MUT) were obtained from the RCSB-PDB database. The X-ray crystallographic structures show mutations at the conserved sites G20 and C92 [8].

### Molecular Docking:

Autodock 4.2 docking tool was used for RNA-ligand interaction study and their effect on the binding affinity due to various mutations on the key nucleotides in the binding pocket [9]. ADT tools and Chimera platform were used for the preparation of ligand and receptor (RNA) files as well as interaction analysis [10]. The Macromolecule File-The downloaded PDB files were first read in chimera visualization tool then the water molecules, protein chain and heteroatoms were removed, and the macromolecule was subjected to Dock prep wizard. Polar hydrogen atoms and amber force field potential charges (AMBER ff99bsc0) were added to standard nucleotides while for non-standard nucleotides AM1-BCC charge system was calculated using antechamber program. Then the macromolecule was added to ADT tools GUI after conversion of mol2 file to. pdbqt file using Open Babel software. The Ligand File-The ligand PDB files were generated using Chimera tool then they were subjected to ADT where all hydrogens were merged, and Gasteiger charges were added. ADT then determined the best root. Eight torsion angels were calculated. The ligand file was then saved with a ligand.pdbqt extension. Preparation of the Grid Parameter File-A three-dimensional box (grid) was created at the catalytic pocket nucleotide co-ordinates obtained from crystallographic pdb file. Since the ligand can take any conformation, the grid box generated should be large enough in volume to allow any free rotation of ligand, even if it is in fully extended conformation. The parameters required to create such a grid are stored in the grid parameter file: molecule.gpf. Preparation of Docking Parameter File-The docking parameter file moves the ligand molecule in the vicinity of grid box using the map files and other properties defined for the ligand. We used genetic algorithm as search method with 50 runs and 300 population size. After the preparation of required file autodock job was run and the docked ligand files (.dlg extension) were used for study.

### Molecular dynamics simulation:

The MD simulations were carried out for wild type (3IRW) and mutant (3MUT) riboswitch-ligand complexes using the GROMACS 2019.6 package and classical AMBER force field parameters [11]. The ligand force field parameters were generated using ACPYPE/Antechamber program [12]. The protocol for all MD simulations is described as follows: the force field parameterized riboswitch-ligand complex was enclosed in a cubical box at a minimum distance of 10 Å from the complex surface to the edges of the box. The box was solvated using TIP3P water model and counter-ions were added to electrically neutralize the whole system. To remove unfavorable contacts 5000 steps of steepest-descent energy minimization was carried with maximum force convergence being less than 100 kcal mol-1 nm-1 [13]. Then each of the energy-minimized structure was equilibrated: 100 ps using canonical (NVT) and 100 ps using isothermal-isobaric (NPT) ensembles. During equilibration, each system was coupled with the Berendsen thermostat [14] and Parrinello-Rahman barostat [15], respectively to maintain temperature 300 K and pressure 1 bar, while the positions of c-di-GMP molecule and riboswitch were restrained. Finally, an unrestrained MD production run of 100 ns was carried out under NPT ensemble. For each simulation, an integration step of 2 fs was used. Particle Mesh Ewald summation method was used for the calculation of long-range electrostatic interactions [16]. The cut-off distance used for non-bonded interactions was 10 Å. The LINC algorithm was used to restrain the bonds containing hydrogen atoms [17]. The MD production run uses Leapfrog algorithm [18].

### Binding free energy calculations:

To calculate the binding energy of the two complexes Molecular Mechanics/Poisson-Boltzmann surface area (MM/ PBSA) method was used [19]. For each 100 ns MD trajectory 1000 snapshots at an equal interval of 100 ps was used. This method is widely used for free energy calculation from MD trajectory. The per-residue energy contribution was also computed to understand the contribution of individual amino acids to the total binding energy.

## Results and Discussion:

The molecular docking data shows that the native/wild type (3IRW) has minimum binding energy followed by 3MUM and 3MUT mutant types respectively, elucidating the most favourable binding of ligand to catalytic pocket (Table 1 - see PDF). A previous study reported that there should be loss in binding affinity/binding free energy in mutated structures [8]. Among the mutant complexes, the mutant 3MUT shows least binding energy showing that the two mutations (G20A_C92U) is more favourable and similar to wild type than single mutations (G20 or C92). It was reported that the mutant 3MUT binds effectively to c-di-AMP instead of c-di-GMP due to two mutations at the conserved residues [8]. Similarly, different analogs of c-di-GMP ligand can also bind to the c-di-GMP riboswitch with different affinities elucidating flexibility in riboswitch-ligand binding [20]. The experimental studies (i.e., the dissociation constant KD value data) revealed that the mutated structures have high KD values as compared to the native riboswitch [8]. Therefore, this study also showed that the mutated structures have less affinity towards their ligand. Thus free energy results are in consistent with the experimental data and show a pattern in selectivity of c-di-GMP ligand for different mutation at catalytic pocket.

**Figure 1:**
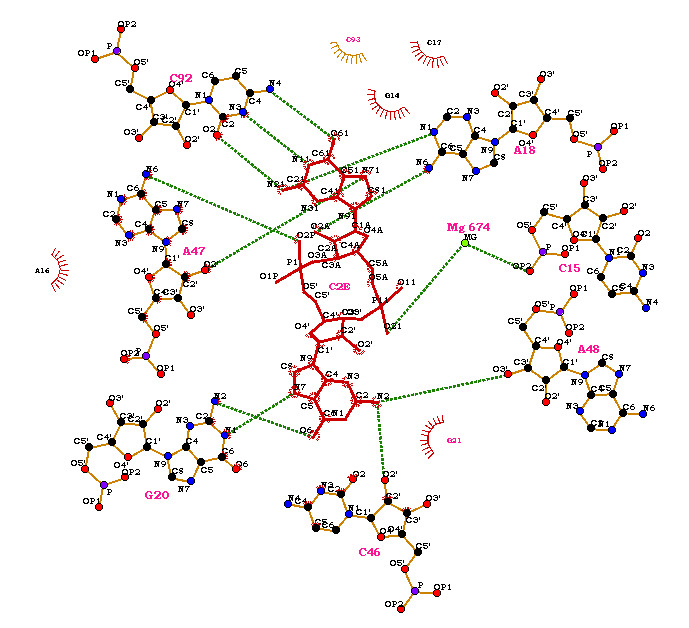
Schematic diagram of interaction of ligand with the receptor catalytic pocket residues for wild type. The green colour shows the hydrogen bonds and the red stick model shows the ligand molecule while the receptor residue atoms are shown in different colours [red for oxygen atoms; blue for nitrogen atoms; black for carbon atoms]

**Figure 2:**
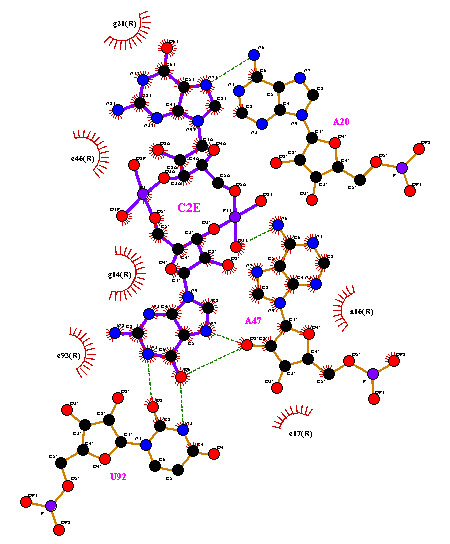
Schematic diagram of interaction of ligand with the receptor catalytic pocket residues for mutant G20A/C92U.

The interaction diagram generated by LigPlot plus shows a total of eleven (11) hydrogen bonds with the atoms among the ligand and residues (Figure 1). Similarly other mutated structures were also introspected for the hydrogen bond interactions. The number of hydrogen bonds for 3mut is six, showing that the strength of binding decreases due to mutation (Figure 2). The nucleotide A47 is highly conserved in riboswitch structure, this shows 2 hydrogen bond with c-di-GMP (c2e) ligand while in mutated structure this number increases to 3 thus this nucleotide is pivotal, as in case of mutation its oxygen atom forms an extra hydrogen bond.

**Figure 3:**
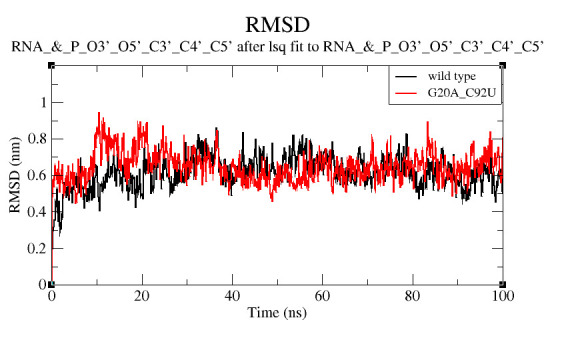
RMSD plot of backbone atoms (P, O3', O5', C3', C4', C5') with the first equilibrated structure as reference for entire MD simulation. Black colour represents the trajectory of wild type (3IRW) while the red trajectory shows G20A_C92U mutant (3MUT).

**Figure 4:**
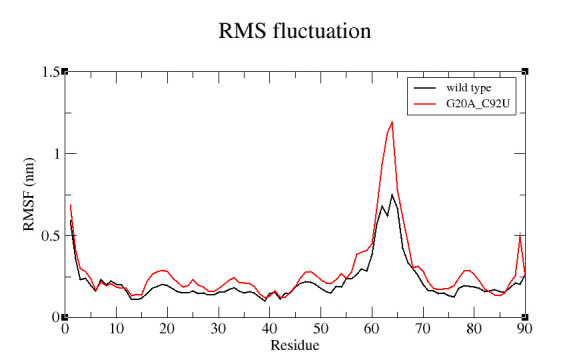
RMSF of atoms C1' in nucleotides of complexes Vs residue number: black for wild type and red for mutant type.

**Figure 5:**
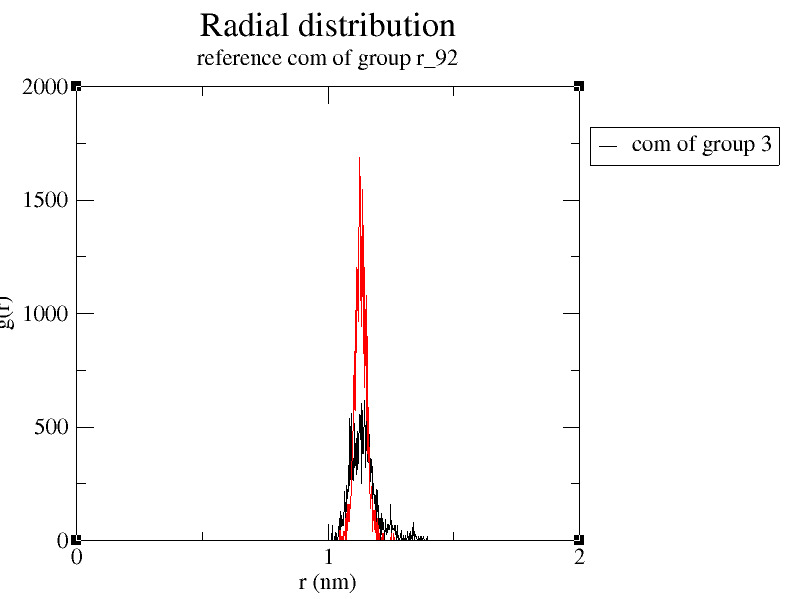
RDF comparative graph for ligand from wild type C92 (black) and mutant U92 (red).

**Figure 6:**
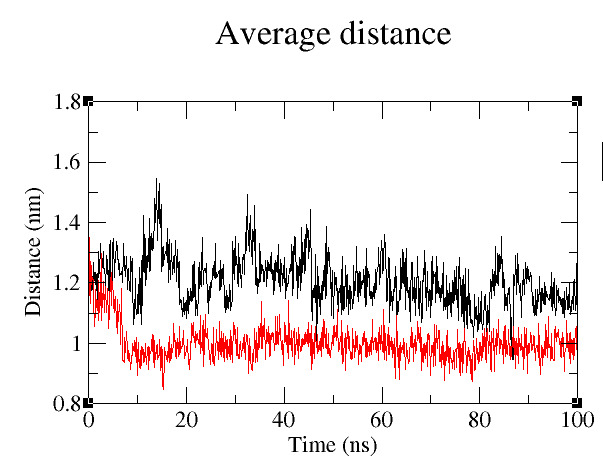
Distance graph for both wild type (black) and mutant (red) between G20 and C92 for entire MD time.

The binding of c-di-GMP to the wild type and the mutated riboswitches was illustrated using MD simulations [21,22]. The stability and flexibility of the aptamer-ligand complex for both wild type and mutated complexes were studied by plotting the RMSD (root mean square deviation) and RMSF (root mean square fluctuation) graphs for 100ns trajectories. RMSD of backbone atoms (P, O3', O5', C3', C4', and C5') were computed relative to the first equilibrated structure through the entire MD simulation (Figure 3). It was observed that the systems reached the equilibrium after 40ns. The average RMSDs of both the complexes show a minor difference of 0.034 nm as both are similar structure (wild type RMSD: 0.614 nm and mutant RMSD: 0.648 nm) with only two nucleotides difference where a purine (G) is replaced by another purine (A) and a pyrimidine (C) is replaced by another pyrimidine (U). Similarly, Luo et al. (2014) also showed that complexes binding to the c-di-GMP ligand and their analogs are structurally alike but they might differ in their unbound state, supporting the RMSD results [23]. The flexibility of the complexes illustrated by the fluctuation of the C1' atoms of each nucleotide residues over 100ns MD run is shown by Figure 4. The graph shows the mutant complex is more flexible compared to the wild type and higher fluctuations for all the residual atoms. This indicates that mutation affects the flexibility of atoms. Interestingly, the catalytic pocket residues do not show any fluctuation for both the complexes while the critical residue U92 (for mutant) shows lower fluctuations than the wild type C92. This show that the catalytic pocket C1' atoms that is the junction nucleotide atoms were stable, while the P1, P2 and P3 helices for mutant riboswitch aptamer shows flexibility. The region near 60 to 70 residues shows a sharp peak for mutant as this is the loop region of P3 helix and very flexible as it is free to move in space. Thus RMSF result illustrates more flexible mutant aptamer with relatively stable binding pocket. The radial distribution function (RDF) of ligand about U92 as reference for mutant type shows a high probability of finding the ligand molecule near U92 indicating interaction among both the entities as compared to wild type (Figure 5). This observation supports the RMSF results where the critical residue U92 (for mutant) shows lower fluctuations than the wild type C92. The average distance between G20 and C92 for wild type is 1.20 Å while the average distance between A20 and U92 for mutant type is 0.99 Å. The average distance difference clearly indicates that the mutant type distance decreases showing that the stacking of A47 becomes difficult but the distance in the wild type easily allows base stacking (Figure 6).

**Figure 7:**
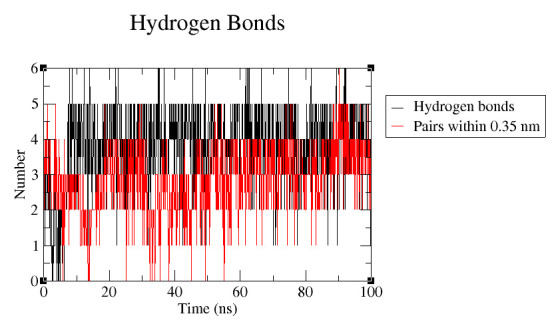
Number of hydrogen bonds between ligand and G20_C92 (wild type, black); ligand and A20_U92 (mutant, red) for entire MD run.

The effect of mutation on the binding of c-di-GMP ligand with riboswitch aptamer was analysed. The hydrogen bond formation between the target nucleotides and the ligand were plotted (Figure 7). This plot indicated loss of hydrogen bonds for mutant complex elucidating decrease in the binding affinity. The average number of hydrogen bonds per timeframe for wild type is 3.79 while that for mutant is 2.84. The result is in accordance with the docking and LigPlot results. Binding free energy of both the complexes was calculated using MM/PBSA method using 500 frames extracted at an equal intervals of 200 ps for 100 ns MD trajectories [19]. This method has been widely applied in various studies such as stability, target-ligand binding interactions and drug designing [24,25]. The MM/PBSA approach is also used to investigate residual binding energies in molecular recognition processes as it effectively states the contribution of each residue in binding process [26]. The contributions of different interactions were either positive or negative to the overall binding free energy and summarized in Table 2 (see PDF). The wild type complex showed a highly negative binding energy (-425.111 kJ mol-1). The components of various energy terms contributing to the total binding energy results revealed that due to high positive polar solvation energy, the binding strength of c-di-GMP ligand to the riboswitch aptamer decreases significantly (Table 2 - see PDF). Among the various interactions, van der Waal energy (ΔEvdW) showed the most favourable contributions towards the negative binding free energy of the complex.

**Figure 8:**
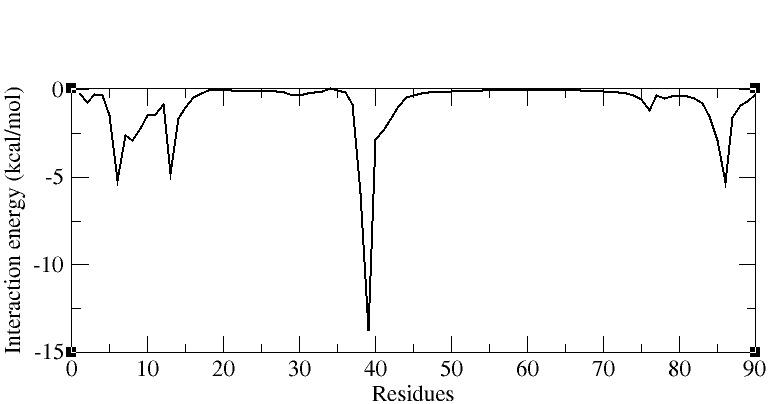
Per residue binding free energy spectrum for wild type complex

The individual contribution of each nucleotide to the binding energy of wild type complex, per-residue interaction energy profile was plotted (Figure 8). The graph revealed five key residues G14, G21, C46, A47, and C92, which are critical to binding of ligand with high affinity rendering stability to the complex. Li et al (2019) also elucidated the role of these key residues in the allosteric change between the unbound and bound state of the c-di-GMP I riboswitch with the c-di-GMP ligand, thereby supporting the above observation [27]. The most favourably contributing residue was A47 having a negative binding energy of -13.30 kcal/mol. The A47 residue is base stacked in between the two guanines of c-di-GMP ligand and is phylogenetically conserved in all the c-di-GMP I riboswitches; if this residue is mutated then the c-di-GMP ligand does not get bind to its riboswitch aptamer.

The binding free energy data for the mutant type is highly positive showing least binding affinity that is energy is needed to induce binding of ligand to the aptamer. The experimental results have also shown that KD value for G20A_C92U was found to be 4900+/-960 nM, which was very high, compared to 0.011 nM for wild type [7]. The different energy contributions to the binding free energy gave the insight that due to a very high positive electrostatic energy contribution (ΔEelec), the total binding energy turned out to be positive. This energy distribution and binding free energy needs to be explored further using other methods such as FEP (free energy perturbation method), TI (thermodynamic integration) and MM-GBSA (molecular mechanics generalized Born surface area).

## Conclusion:

This study provides molecular and dynamical insights to understand the binding interaction of riboswitch aptamer and ligand with the effect of induced mutations at binding/catalytic points. Information on the binding of docked ligand to the mutated conserved G20 and C92 residues of c-di-GMP I riboswitch using molecular dynamics simulation is important. The result shows that the mutations affected the riboswitch-ligand interactions. The base stacking of critical nucleotide A47 is responsible for the stable binding of c-di-GMP to c-di-GMP I riboswitch which is affected in the mutant complex due to decrease in distance between A20 and U92. The residues G14, G21, C46, A47 and U92 were identified as the key residues which contributed effectively to the binding of c-di-GMP I riboswitch with the natural ligand.
